# 3D Whole‐heart free‐breathing qBOOST‐T2 mapping

**DOI:** 10.1002/mrm.28039

**Published:** 2019-10-21

**Authors:** Giorgia Milotta, Giulia Ginami, Aurelien Bustin, Radhouene Neji, Claudia Prieto, René M. Botnar

**Affiliations:** ^1^ School of Biomedical Engineering and Imaging Sciences King's College London London United Kingdom; ^2^ MR Research Collaborations Siemens Healthcare Limited Frimley United Kingdom; ^3^ Escuela de Ingeniería Pontificia Universidad Católica de Chile Santiago Chile

**Keywords:** 3D whole‐heart, black‐blood imaging, bright‐blood cardiac anatomy, respiratory motion correction, T2 mapping

## Abstract

**Purpose:**

To develop an accelerated motion corrected 3D whole‐heart imaging approach (qBOOST‐T2) for simultaneous high‐resolution bright‐ and black‐blood cardiac MR imaging and quantitative myocardial T2 characterization.

**Methods:**

Three undersampled interleaved balanced steady‐state free precession cardiac MR volumes were acquired with a variable density Cartesian trajectory and different magnetization preparations: (1) T2‐prepared inversion recovery (T2prep‐IR), (2) T2‐preparation, and (3) no preparation. Image navigators were acquired prior the acquisition to correct for 2D translational respiratory motion. Each 3D volume was reconstructed with a low‐rank patch‐based reconstruction. The T2prep‐IR volume provides bright‐blood anatomy visualization, the black‐blood volume is obtained by means of phase sensitive reconstruction between first and third datasets, and T2 maps are generated by matching the signal evolution to a simulated dictionary. The proposed sequence has been evaluated in simulations, phantom experiments, 11 healthy subjects and compared with 3D bright‐blood cardiac MR and standard 2D breath‐hold balanced steady‐state free precession T2 mapping. The feasibility of the proposed approach was tested on 4 patients with suspected cardiovascular disease.

**Results:**

High linear correlation (y = 1.09 × −0.83, R^2^ = 0.99) was found between the proposed qBOOST‐T2 and T2 spin echo measurements in phantom experiment. Good image quality was observed in vivo with the proposed 4x undersampled qBOOST‐T2. Mean T2 values of 53.1 ± 2.1 ms and 55.8 ± 2.7 ms were measured in vivo for 2D balanced steady‐state free precession T2 mapping and qBOOST‐T2, respectively, with linear correlation of y = 1.02x+1.46 (R^2^ = 0.61) and T2 bias = 2.7 ms.

**Conclusion:**

The proposed qBOOST‐T2 sequence allows the acquisition of 3D high‐resolution co‐registered bright‐ and black‐blood volumes and T2 maps in a single scan of ~11 min, showing promising results in terms of T2 quantification.

## 1 INTRODUCTION

Cardiac MR (CMR) is a powerful tool for the assessment of a wide range of pathologies such as congenital heart disease, coronary artery disease, myocardial inflammation and edema.^1^, ^2^, ^3^ However, several CMR sequences with different acquisition planning and geometries are needed to assess these pathologies. In particular, bright‐blood imaging can be used to visualize whole‐heart anatomy and the great thoracic vessels.^4^ Black‐blood imaging provides visualization of atrial/ventricular myocardial, aortic and pulmonary wall and enables thrombus/hemorrhage detection.^5^ T2 mapping enables noncontrast quantitative tissue characterization, with increased myocardial T2 values reported to correlate with edema that can be associated with acute myocardial infarction,^6^, ^7^ cardiomyopathies^8^, ^9^ and transplant rejection.^10^

Bright‐blood CMR angiography (CMRA) for coronary and whole heart anatomy visualization is conventionally performed free‐breathing with 1D diaphragmatic navigator (dNAV) gating.^11^ Similarly, thrombus/hemorrhage visualization is typically performed with a 3D free‐breathing noncontrast enhanced black‐blood T1‐weighted inversion recovery (IR) technique^5^ with 1D dNAV. 1D navigator gating approaches minimize respiratory motion by acquiring data only when the navigator signal is within a small gating window (~5‐6 mm), leading to long and unpredictable scan times. To enable shorter and more predictable scan times several self‐gating techniques have been proposed to directly track and correct for the respiratory motion of the heart.^12^, ^13^, ^14^, ^15^, ^16^, ^17^, ^18^ Conventional cardiac T2 maps are acquired with T2 prepared balanced steady‐state free precession (bSSFP) in 2D short‐axis views, under several breath‐holds, requiring patient cooperation and expert planning. T2 preparation (T2prep) pulses with increasing T2prep durations are used to acquire several T2‐weighted images that follow an exponential T2 decay curve.^19^, ^20^, ^21^ A pause time of several cardiac cycles is used to allow for T1 recovery before applying the next T2 prepared imaging series.^3^ Typically, only a single 2D slice can be acquired for each breath hold leading to limited spatial resolution and coverage. High‐resolution free breathing 3D T2 mapping of the heart has been demonstrated using 1D dNAV but leads to long and unpredictable scan times,^20^ hindering the acquisition of high isotropic resolution images. 1D dNAVs have also been used to correct for foot‐head translational respiratory motion with ~100% scan efficiency,^21^ enabling shorter scan times; however, the heart is not directly tracked with this approach and a motion model to relate the diaphragmatic to cardiac motion is needed. 1D respiratory self‐navigation has been investigated for 3D radial trajectories, enabling the acquisition of 1.7 mm isotropic T2 maps in ~18 min.^22^ However, acquisition time (TA) remains a challenge with this approach because a heart beat is necessary between acquisitions to allow magnetization recovery.

Furthermore, the sequences (bright‐blood, black‐blood, and T2 mapping) are usually performed sequentially, with different geometries (2D and 3D) and orientations, and under different breathing conditions (i.e., breath‐hold and free‐breathing), leading to prolonged TAs and potential miss‐registration errors between the images. To partially overcome this problem, a T2 prepared Bright‐blood and black‐blOOd phase SensiTive (BOOST) IR sequence^23^ has been recently proposed to provide respiratory motion compensated and co‐registered bright‐ and black‐blood 3D whole‐heart images. Nevertheless, this sequence is unable to provide quantitative tissue characterization and still requires long scan times (~20 min with fully sampled acquisitions).

The aim of this work was to develop a novel accelerated and respiratory motion compensated 3D whole‐heart sequence (qBOOST‐T2), which provides co‐registered high‐resolution 3D bright‐blood, black‐blood, and quantitative T2 map volumes from a single free‐breathing scan of ~11 min. This was achieved by extending the BOOST sequence^23^ to enable undersampled acquisition and to provide high‐resolution 3D whole‐heart T2 maps. The proposed sequence is based on the acquisition of 3 interleaved datasets with different magnetization preparation pulses. The first volume provides bright‐blood anatomy visualization, the black‐blood volume is obtained by means of phase sensitive IR (PSIR), ‐like reconstruction^24^ between the first and third datasets, and T2 maps are generated by matching the signal evolution to a simulated dictionary.

## 2 METHODS

### 2.1 qBOOST‐T2 framework

**Figure 1:**
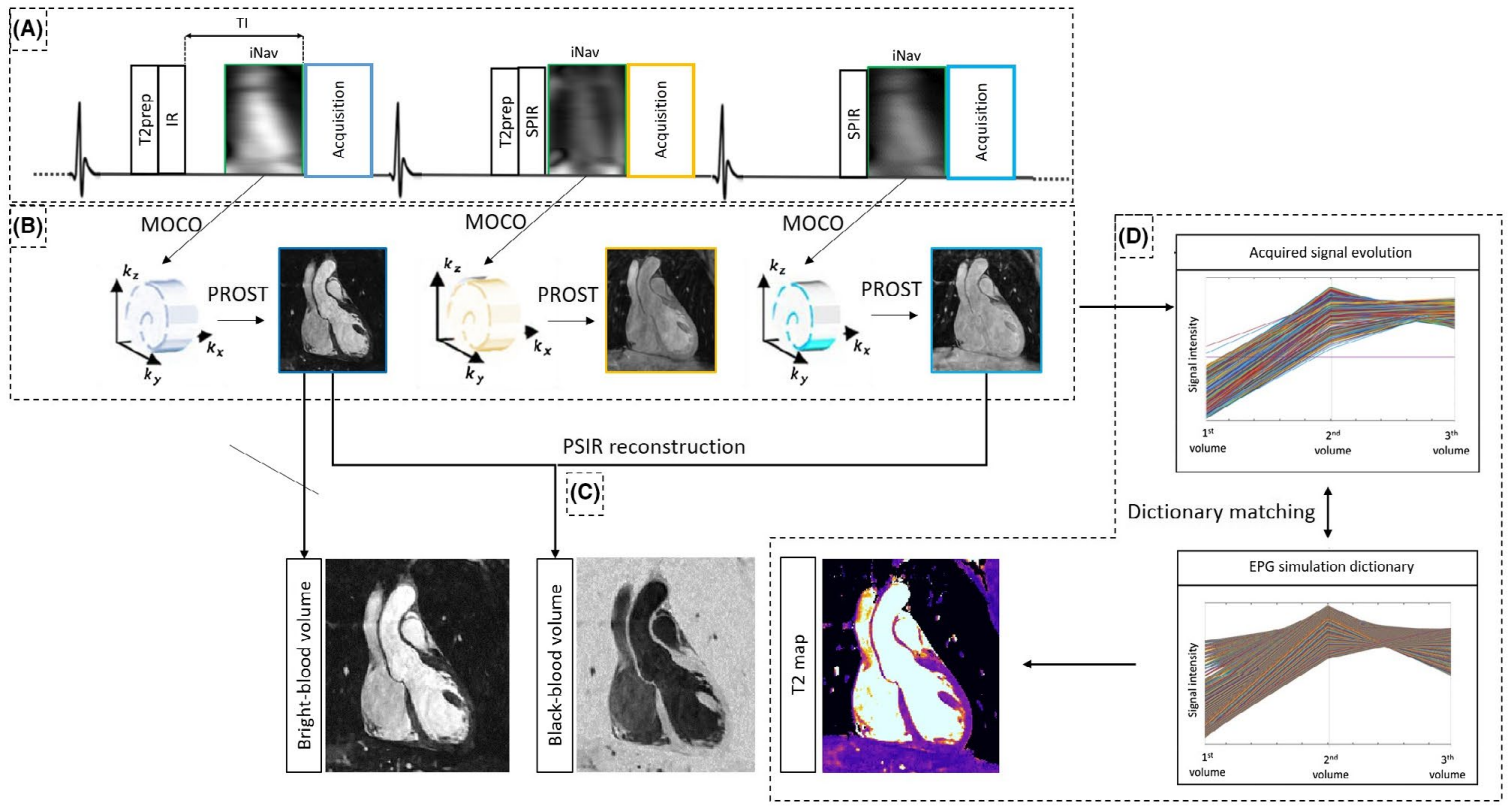
Framework of the proposed 3D whole‐heart qBOOST‐T2. Acquisition (A), Three undersampled interleaved bSSFP bright‐blood volumes are acquired with: (1) T2prep‐IR, (2) T2prep, and (3) no preparation modules, respectively. 2D‐iNAVs are acquired in each heartbeat before image acquisition. Reconstruction (B), image navigators are used to estimate/correct SI and LR translational motion. Translational beat‐to‐beat motion correction is performed on the 3 datasets independently and each volume is reconstructed with 3D PROST reconstruction. PSIR reconstruction (C), Black‐blood images are obtained by performing a PSIR reconstruction between the dataset acquired with T2prep‐IR preparation (bright‐blood image) and the third volume as a phase reference. T2 map generation (D), T2 map is generated by matching the measured signal and a previously generated EPG simulated dictionary. The first dataset acquisition includes a STIR fat suppression (TI = 110 ms), whereas the second and third datasets use a SPIR pulse for fat saturation

The proposed 3D whole‐heart electrocardiograph triggered qBOOST‐T2 mapping sequence is shown in Figure 1. Three interleaved bright‐blood bSSFP volumes were acquired with an undersampled variable density Cartesian trajectory with spiral‐like profile order.^24^, ^25^ A nonselective T2prep‐IR module with T2prep length = 50 ms and TI = 110 ms was applied before the first dataset acquisition. T2 preparation (T2prep length = 30 ms) was performed before the second volume, whereas the third dataset is acquired with no preparation. Fat suppression was achieved with a short inversion time IR (STIR) approach^26^ in the first dataset, whereas spectral presaturation fat suppression (SPIR, spectral presaturation IR)^27^ was used in the second and third datasets.

2D low‐resolution iNAVs were acquired before the acquisition of each volume to estimate and correct for superior‐inferior (SI) and left‐right (LR) translational respiratory motion, enabling 100% respiratory scan efficiency. A template‐matching algorithm with a mutual information similarity measure^28^ was used to estimate SI and LR beat‐to‐beat translational motion from the iNAVs. Outliers due to deep breaths (outside the interval calculated as mean ± 2 standard deviations) were removed and 2D translational motion correction is performed as a linear phase shift in k‐space.^29^

Each undersampled translational motion corrected 3D volume was independently reconstructed with a 3D low‐rank patch‐based reconstruction (3D‐PROST).^25^ PROST undersampled reconstruction exploits local (within a patch) and nonlocal (between similar patches within a neighborhood) redundancies of the 3D volumes in an efficient low‐rank formulation. The reconstruction is formulated as an iterative 2‐step process: (1) a L2‐norm regularized parallel image reconstruction using the denoised volume from step 2 as prior knowledge, and (2) a low‐rank patch based denoising. The first step is solved using conjugate gradient whereas the second step is solved by using a truncated singular value decomposition.

3D affine image registration was performed between the 3 reconstructed volumes. The T2prep‐IR volume provided bright‐blood anatomy visualization, while a PSIR‐like reconstruction^24^ between the first and third acquired volume was performed to obtain the black‐blood dataset. Whole‐heart T2 maps were generated by matching the measured signal evolution of each voxel through the 3 motion corrected and reconstructed volumes to the closest entry of a subject‐specific dictionary obtained by means of extended phase graphs (EPG) simulations.^25^ EPG simulations provide the evolution of transversal and longitudinal magnetization for the given sequence and avoid the use of recovery periods, usually needed for the complete recovery of the longitudinal magnetization. The dictionary generation and the matching step between measured and simulated signal are described in more detail hereafter.

### 2.2 Dictionary generation and matching

EPG simulations were carried out to generate a subject‐specific dictionary. Trigger delay and acquisition window parameters were specified for each simulation according to the heart rate (HR) and mid‐diastolic resting period of the subject. Taking into account the centric k‐space reordering of the acquisition trajectory, the simulated dictionary was generated considering the mean absolute value of the signal for the k‐space central region (40% of the readouts per heartbeat), containing contrast information. Longitudinal magnetization evolution was used to determine the signal magnetization polarity. The dictionary was generated with 3 different T1 values = (900, 1100, 1300) ms and variable T2 values in the range (minimum: step size: maximum) (4:2:100,105:5:200,210:10:450) ms.^30^ Healthy myocardium value at 1.5T is T1 = 1100 ms^31^; however, additional T1s (900 ms and 1300 ms) were included in the dictionary to account for possible sources of T1 variability. The simulated T2 value range was selected to enable coverage of a wide range of T2s, including healthy myocardium (T2 ~ 50 ms), diseased myocardium (i.e., edema T2 ~ 60 ms), and blood (T2 ~ 250 ms).^3^ Quantitative T2 maps were generated by matching each measured and normalized signal evolution to a specific dictionary entry, corresponding to a unique T2 value. The matching was performed minimizing the least square error between the measured signal and the EPG‐based dictionary entry.

Before matching, 2 PSIR reconstructions were performed between the T2prep‐IR prepared and the nonprepared datasets and between T2‐prepared and nonprepared datasets. These PSIR reconstructions were used to systemically restore signal polarity that would affect the matching with the simulated dictionary. The 3 translational motion corrected volumes were normalized in time by dividing each voxel in each volume by the root mean square of the corresponding voxels in the 3 volumes. The obtained datasets were used to obtain the normalized signal evolution, through the 3 acquired volumes, for each voxel.

### 2.3 Experimental design

The proposed qBOOST‐T2 sequence was tested in simulations, in a T2 phantom, on 11 healthy subjects (5 males; mean age, 29 years; range, 27‐35 years) and on 4 patients with suspected cardiovascular disease (3 males; mean age, 51 years; range, 25‐75 years). Acquisition was performed on a 1.5T MR scanner (MAGNETOM Aera, Siemens Healthcare, Erlangen, Germany) with an 18‐channel chest coil and a 32‐channel spine coil. Written informed consent was obtained from all participants before undergoing the MR scans and the study was approved by the Institutional Review Board.

#### 2.3.1 Simulations

EPG simulations were carried out to investigate the matching sensitivity to long T1 and T2 pairs and the T2 dependency on different simulated HRs. Signal evolution for different T1 and T2 pairs, ranging, respectively, between T1 = (800:100:1400) ms and T2 = (40:6:88) ms were simulated and matched to an EPG dictionary generated with T1 = (900, 1100, 1300) ms and T2 = (4:2:50, 50:5:200, 200:10:450) ms. HR dependency was assessed by matching the signal evolution generated with different T2 = (40:6:88) ms, fixed T1 = 1100 ms and variable HR = (40:20:120) beats per min (bpm) to a dictionary with T1 = (900, 1100, 1300) ms and T2 = (4:2:50, 50:5:200, 200:10:450) ms.

#### 2.3.2 Phantom

Data acquisition was performed in an in‐house developed T2 phantom to test the sequence ability to differentiate between healthy and diseased myocardium (T2_myoc_ = 52 ms and T2_myoc‐diseased_ = 65 ms). The phantom consists of 6 vials with increasing agar concentrations (0.8, 1, 1.5, 2, 3, and 5%).^32^ Spin echo (SE) and IR spin echo (IRSE) experiments were performed to characterize, respectively, the T2 and T1 of the phantom. The SE acquisition parameters included transversal orientation, field of view (FOV) = 280 × 280 × 20 mm^3^, resolution= 2 × 2 × 4 mm^3^, TR = 10 s, TEs = (15, 30, 60, 120, 240, 480) ms and total TA of 2 h and 12 min. The IRSE was acquired with TR = 10 s, TE = 12 ms and TIs = (50, 100, 150, 300, 500, 1000, 2000, 3000) ms in a total TA = 6 h and 16 min. Reference T2 and T1 values obtained with the SE and IRSE experiments are shown in Supporting Information Table S1, which is available online.

Acquisition parameters for qBOOST‐T2 included: bSSFP acquisition with centric k‐space ordering, transversal orientation, resolution = 1 × 1 × 2 mm^3^, FOV = 280 × 280 × 20 mm^3^, TI = 110 ms, T2prep_1st‐volume_ = 50 ms, T2prep_2nd‐volume_ = 30 ms, flip angle (FA) = 90 degrees, simulated HR = 60 bpm, trigger delay = 700 ms, TE/TR = 1.57/3.6 ms, bandwidth = 822 Hz/pixel, 14 start‐up echoes for iNAVs acquisition, 30 segments per heart beat corresponding to an acquisition window of 110 ms, and acceleration factor of 4 for each volume leading to a total scan time ~3 min. The conventional T2 prepared 2D T2 map was acquired with a bSSFP sequence with FA = 70 degrees, FOV = 290 × 360 mm^2^, resolution = 1.8 × 1.8 mm^2^, slice thickness = 8 mm, TE/TR = 1.18/3.5 ms, bandwidth = 1185 Hz/pixel, simulated heartbeat = 60 bpm, trigger delay = 700 ms, T2prep preparations = (0, 28, 55) ms, 3 recovery heart beats and linear k‐space ordering.^3^ Three slices were acquired in transversal direction with acquisition time of 12 heart beats per slice. Additionally, phantom acquisitions were performed with different HRs = (40:20:120) bpm to investigate the HR dependency on T2 quantification using qBOOST‐T2.

#### 2.3.3 Healthy subjects

Data were acquired with the proposed qBOOST‐T2, conventional 2D bSSFP T2 mapping, and 3D CMRA sequence with iNAV‐based respiratory motion correction^14^ for comparison purposes. Imaging parameters for qBOOST‐T2 included: coronal orientation, resolution= 1 × 1 × 2 mm^3^, FOV = 320 × 320 × 96‐104 mm^3^, TI = 110 ms, T2prep_1st‐volume_ = 50 ms, T2prep_2nd‐volume_ = 30 ms, FA = 90 degrees, TE/TR = 1.57/3.6 ms, bandwidth = 822 Hz/pixel, 14 start‐up echoes for iNAVs acquisition, and acceleration factor of 4 for each volume leading to a total scan time 11 ± 1.2 min. The 3D CMRA dataset was acquired with a fully sampled bSSFP sequence and imaging parameters matching the qBOOST‐T2 acquisition with a single T2prep of 40 ms, resulting in a total scan time 11.5 ± 1.4 min. Imaging parameters for the standard 2D T2 mapping were set as follows: resolution = 1.8 × 1.8 mm^2^, slice thickness = 8 mm, FOV = 290 × 360 mm^2^, FA = 70 degrees, TE/TR = 1.18/3.5 ms, bandwidth = 1185 Hz/pixel, T2prep preparations = (0, 28, 55) ms with 3 heart beats for magnetization recovery, 3 short axis slices (base, mid, apex) were acquired in a 10 heart‐beats breath‐hold per slice. All acquisitions were electrocardiograph triggered and performed during mid‐diastolic resting period. Trigger delay ranged between 548 and 950 ms and acquisition window ranged between 85 and 125 ms corresponding to 24‐34 segments acquired per heartbeat. The acquisition parameters of the different used sequences are summarized in Supporting Information Table S2.

#### 2.3.4 Patients

The feasibility of the proposed qBOOST‐T2 sequence was tested on 4 patients with suspected cardiovascular disease. Imaging acquisition parameters matched the healthy subject scans. The patients were, respectively, 25, 75, 41, and 63 years old with an average HRs of 45, 72, 85, and 76 bpm. A conventional 2D bSSFP T2 prepared mapping sequence was acquired for comparison purposes with the same imaging parameters used for the healthy subject study.

### 2.4 Reconstruction

2D T2 maps were reconstructed in‐line using the scanner software (Syngo MR E11A, Siemens Healthcare, Erlangen, Germany). Nonrigid motion correction to compensate for in‐plane motion between 2D T2 weighted images and exponential pixel‐wise fitting were performed in‐line on the scanner.

qBOOST‐T2 and CMRA raw data were exported from the scanner and reconstructed in MATLAB (The MathWorks, Inc., Natick, MA) on a dedicated workstation (16‐core Dual Intel Xeon Processor, 2.3 GHz, 256 GB RAM). Translational motion correction to end‐expiration was performed individually on each qBOOST‐T2 dataset in vivo. The 3 datasets were independently reconstructed using 3D‐PROST, with reconstruction parameters set as suggested in Bustin et al.^25^ Total reconstruction time for each of the 3 datasets was 18 min. The T2prep‐IR dataset enables bright‐blood anatomical visualization, whereas the black‐blood volume was obtained after PSIR reconstruction between the first and third datasets. Finally, the 3 acquired datasets were normalized, and dictionary matching was performed to obtain the T2 map, as previously described. The averaged time to generate the dictionary was 2 min and 28 s, whereas the averaged matching time for the entire 3D T2 map was 32.4 s, using a classical least square error minimization.

The 2D translational motion correction to end‐expiration was performed on the fully sampled CMRA dataset and a sensitivity‐weighted coil combination was performed.^33^

### 2.5 Data analysis

#### 2.5.1 Phantom

Conventional 2D bSSFP T2 map and 3D qBOOS‐T2 were compared in terms of accuracy with respect to the SE reference. T2 dependency of dictionary T1 was evaluated by matching the measured signal to 3 different dictionaries: T1s 1st dictionary = (900, 1100, 1300) ms, T1s 2nd dictionary = (900, 1100, 1300, 1600, 1800) ms, and T1s 3rd dictionary = (900, 1100, 1300, 1600, 1800, 2000, 2400, 2600) ms. HR dependency was assessed by performing different acquisitions with HR = (40:20:120) bpm.

#### 2.5.2 Healthy subjects

Quantitative analysis was performed for the 3D T2 maps generated with qBOOST‐T2 and the conventional 2D T2 mapping sequence. 3D T2 maps from qBOOST‐T2 were reformatted to the same slice position as the corresponding 2D T2 maps. Mean T2 values were measured for both sequences by selecting a region of interest (ROI) in the myocardial septum. The standard deviation of the T2 measurements within the ROI was used to quantify the precision of the techniques. Additionally, a Bland Altman analysis was performed to evaluate the agreement between the proposed qBOOST‐T2 mapping technique and the conventional 2D T2 mapping approach.

The American Heart Association 17‐segment model^34^ was used to evaluate the percentage of variation of mean T2 and T2 precision between 2D bSSFP and 3D qBOOST‐T2. The myocardial T2 values of the whole ventricle were measured in 16 American Heart Association segments in 3 slice positions: basal, mid and apex. The 17th segment was excluded from the analysis as the coverage of the reference 2D T2 map was not sufficient to visualize the apical cap. The percentage errors of variation were calculated for each segment and each subject as:

$$T2_{Mean\_variation}=\left(T2_{Mean\_qBOOST}-T2_{Mean\_bSSFP}\right)/T2_{Mean\_bSSFP}\times 100$$

$$T2_{Std\_variation}=\left(T2_{Std\_qBOOST}-T2_{Std\_bSSFP}\right)/T2_{Std\_bSSFP}\times 100$$

The percentage errors of variation were averaged across subjects and displayed as bull's eye plots and bar plots. The T2 homogeneity in the whole left ventricle was evaluated for a representative healthy subject by generating a histogram of per‐pixel T2 values and quantifying the T2 distribution through different coronal slices.

#### 2.5.3 Patient

Mean and standard deviation in T2 quantification were evaluated and compared with conventional 2D bSSFP T2 mapping by selecting a ROI in the septum of the myocardium in apical, mid and basal short axis slices. The American Heart Association 17‐segment model was used to compare the conventional 2D T2 maps and the proposed qBOOST‐T2 mapping in terms of mean T2 value and precision across the whole left ventricle for a representative patient.

## 3 RESULTS

All data acquisitions and reconstructions were carried out successfully and results are reported hereafter.

### 3.1 Simulations

**Figure 2:**
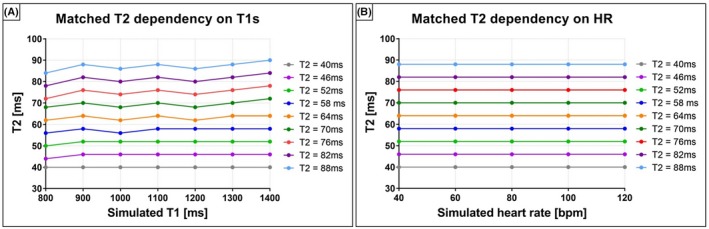
EPG simulations performed to investigate T1 and different HR dependency of the proposed technique. A, Signal evolution of T1/T2 pairs with T2 = (40:6:88) ms and T1 = (800:100:1400) ms were matched to a EPG dictionary with T1 = (900, 1100, 1300) ms. A T2 variability < 5% was observed for all the different T2 values. B, T2 values matched for different simulated HRs. The matched T2 is insensitive to HR variability in simulations experiments

EPG simulation results are shown in Figure 2. A T2 variability < 5% was observed for each simulated T2 value for T1 ranging between 800 and 1400 ms (Supporting Information Figure S1). No T2 variation was observed as function of different HRs.

### 3.2 Phantom

**Figure 3:**
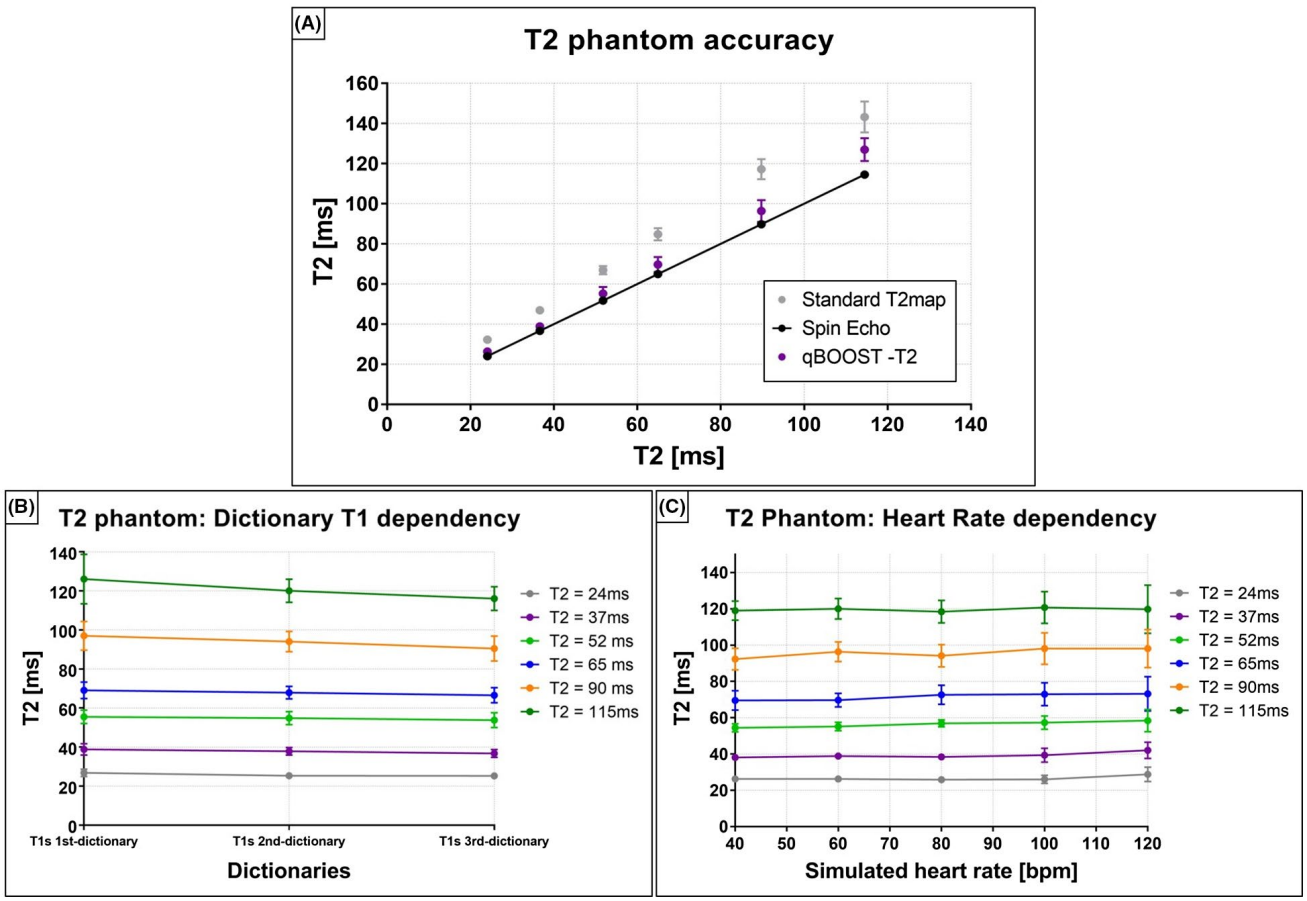
Phantom experiments. A, T2 quantification obtained with reference SE experiment, 2D standard bSSFP T2 mapping and the proposed qBOOST‐T2 sequence for 6 vials with different agar concentration. B, T2 dependency of the proposed sequence to different T1 values included in the dictionary. 1st T1 dictionary = (900, 1100, 1300) ms, 2nd T1 dictionary = (900, 1100, 1300, 1600, 1800) ms, and 3rd T1 dictionary = (900, 1100, 1300, 1600, 1800, 2000, 2400, 2600) ms. A variation of 3.2% and 3.8% is observed, respectively, for T2 values that corresponds to healthy myocardium T2myoc = 55 ms and diseased myocardium T2myoc‐diseased = 65 ms, whereas a variation of 8.6% was observed for a long T2 = 115 ms. C, T2 dependency to different simulated HRs. A T2 variation between 8.2% and 11.6% for HR ranging between 40 and 120 bpm was observed for all the phantom vials

The quantified T2 values obtained with reference SE, 2D bSSFP T2 map, and 3D qBOOST‐T2 are shown in Figure 3A. A T2 overestimation is observed with the conventional 2D T2 mapping sequence, especially for high T2 values, although high linear correlation was observed (y = 1.25x + 2.44 with R^2^ = 0.99). A better agreement in T2 quantification was found between qBOOST‐T2 and SE with linear correlation y = 1.09x – 1.67 (R^2^ = 0.99); however, overestimation of long T2 values was observed.

T2 dependency on the T1 dictionary used is shown in Figure 3B. Including additional T1 values improves the dictionary matching accuracy for longer T2 values (corresponding also to longer T1 values) and reduces the standard deviation within a phantom vial. A variation of 3.2% and 3.8% was observed, respectively, for T2 values that correspond to healthy myocardium T2myoc = 52 ms and diseased myocardium T2myoc‐diseased = 65 ms, whereas a variation of 8.6% was observed for a long T2 = 115 ms. However, T1s > 1400 ms are not expected in vivo; therefore, these values were not included in the dictionary used to match T2 values in healthy subject and patient acquisitions to reduce computational time.

The results of the experiments to investigate HR dependency are shown in Figure 3C. A variation in T2 quantification between 8.2% and 11.6% was observed for all the phantom vials. Additionally, T2 matched standard deviation increased at high HR (100 and 120 bpm), particularly for long T2 values.

### 3.3 Healthy subjects

**Figure 4:**
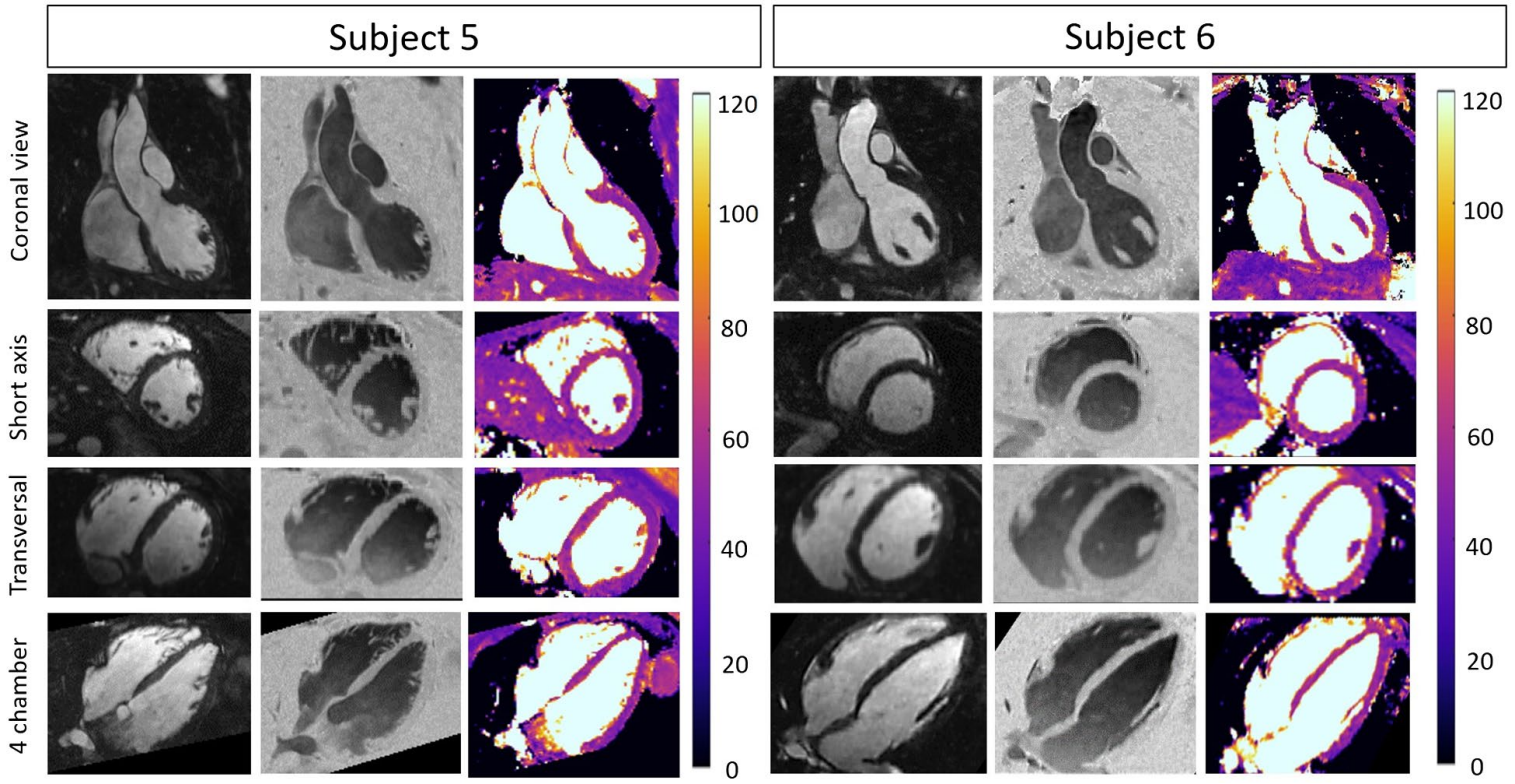
Two representative healthy subjects acquired with the proposed qBOOST‐T2 sequence. 3D high‐resolution bright‐blood (first column), black‐blood (second column), and T2 maps (third column) are co‐registered. Coronal, short axis, transversal, and 4‐chamber views are shown. Acquisition parameters included: 3D bSSFP, T2prep_1st‐heartbeat_ = 50 ms, T2prep_2nd‐heartbeat_ = 30 ms, TI = 110 ms, FA = 90 degrees, resolution = 1 × 1 × 2 mm, 4× undersampling, 14 start‐up echoes for iNAV acquisition

Coronal, transversal, short axis, and 4‐chamber views of 2 representative healthy subjects acquired with the proposed qBOOST‐T2 are shown in Figure 4. Bright‐blood, black‐blood volumes, and T2 maps are shown, respectively, in first, second, and third columns. Atria, ventricles, aorta, and papillary muscles are visible in the anatomical bright‐ and black‐blood images for both subjects. Good left ventricle delineation is observed in the T2 maps of both subjects. Additionally, 3 Supporting Information Videos S1, S2, and S3 show the bright‐blood, black‐blood 3D volumes, and the co‐registered 3D T2 map for 1 representative healthy subject.

**Figure 5:**
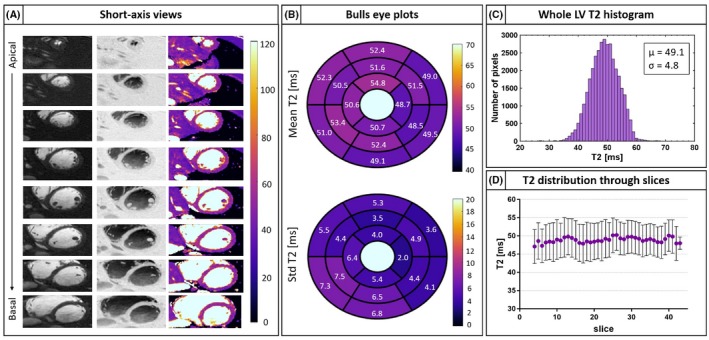
A, Bright‐blood, black‐blood, and T2 map short‐axis views from apex to base are shown for 1 representative healthy subject. The 3D nature of the acquisition permits to obtain complete coverage of the heart. B, Bull's eye plot of average T2 quantification and T2 standard deviation show uniform T2 quantification in all the different segments. C, Histogram of per‐pixel T2 distribution through the whole left ventricle. D, Averaged T2 distribution through coronal slice. Uniform T2 quantification is observed in the left ventricle

Short axis reformatted anatomical bright‐ and black‐blood images and T2 map are shown for a different healthy subject in Figure 5A. The 3D nature of the acquisition allows whole coverage from the apex to the base of the myocardium. Bull's eye plot of mean myocardium T2 quantification and T2 standard deviation are shown in Figure 5B, uniform T2 values are observed across the different segments, although lower precision (corresponding to a higher standard deviation) is observed in the inferior part of the left ventricle. A histogram of per‐pixel T2 distribution is shown in Figure 5C. The mean and standard deviation of T2 distribution were 49.1 ms and 4.8 ms, respectively, whereas maximum and minimum matched T2 values were 71 and 22 ms. Additionally, T2 distribution through coronal slices showed a linear correlation of y = 0.02x + 48.38 (Figure 5D).

**Figure 6:**
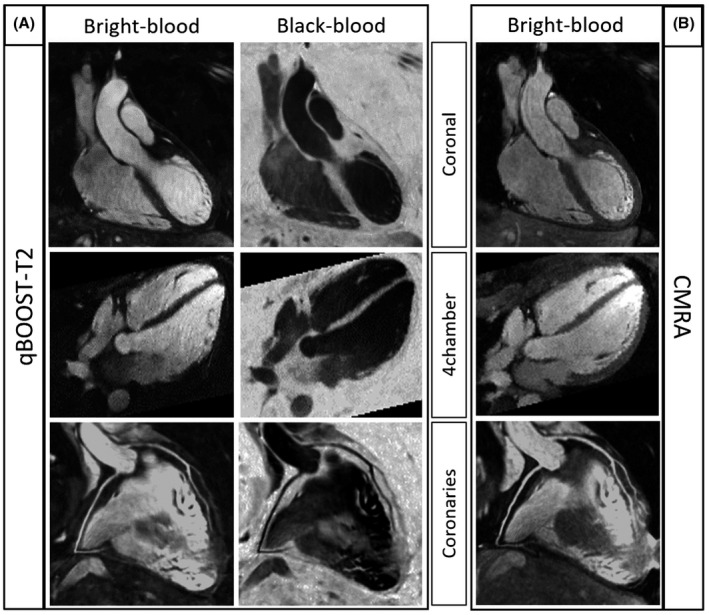
Comparison between bright‐blood anatomical images (first column), black‐blood images (second column) acquired with qBOOST‐T2 (A), and bright‐blood CMRA (B) for 1 healthy subject. Coronal, 4‐chamber views, and coronary artery reformats are shown in first, second, and third row, respectively

Coronal, 4‐chamber views and coronary reformatted images obtained with bright‐blood qBOOST‐T2 and CMRA are shown in Figure 6 for a representative healthy subject. Both approaches show clear delineation of aortic wall, papillary muscles, and coronary arteries.

**Figure 7:**
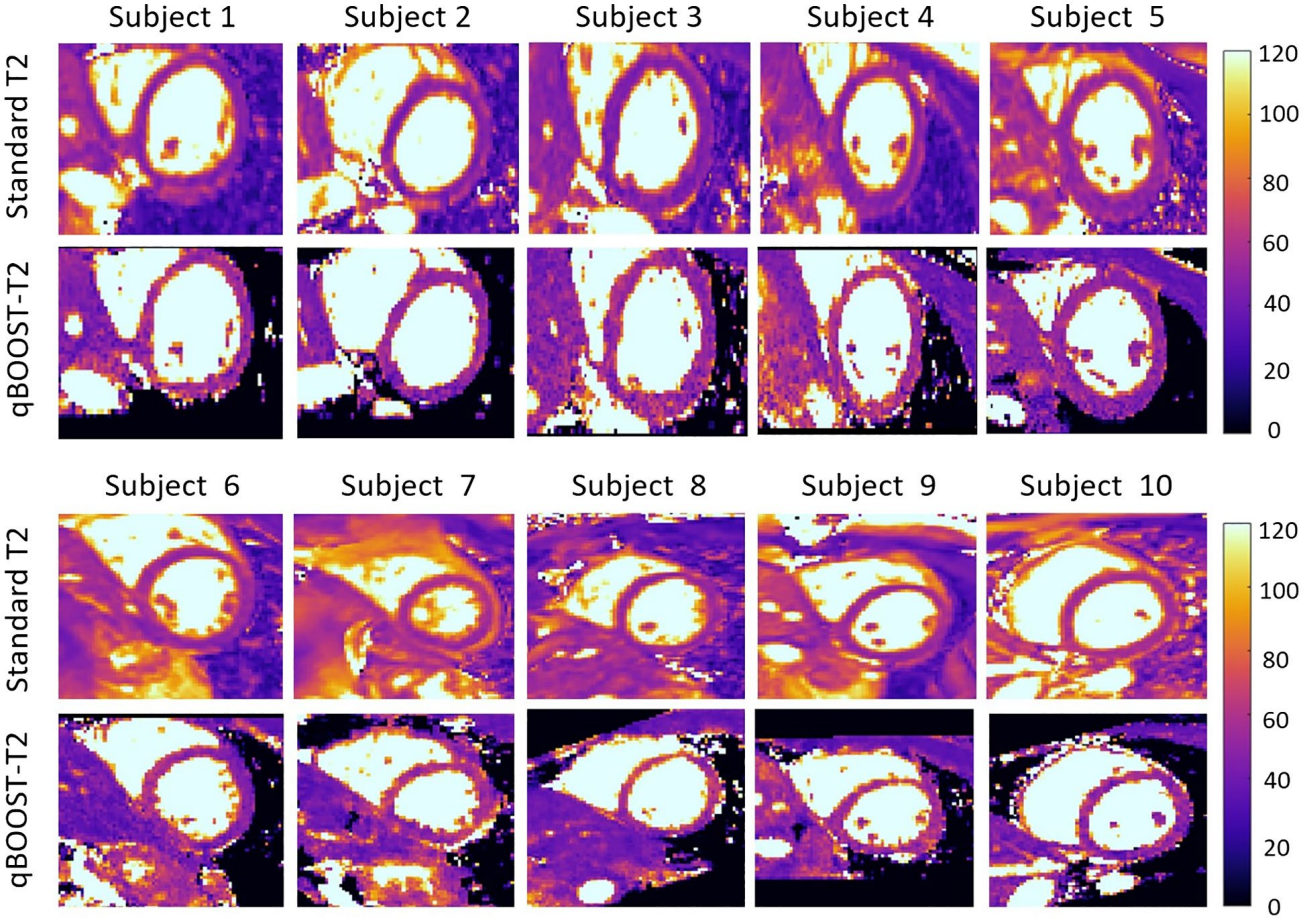
Comparison between 2D short‐axis standard T2 maps and short‐axis reformatted 3D qBOOST‐T2 maps for 10 healthy subjects. qBOOST‐T2 maps have been reformatted to the same slice position of the acquired 2D bSSFP T2 maps. Comparable visual image quality is obtained with the 2 approaches

T2 maps generated with the proposed approach were compared with conventional 2D bSSFP T2 mapping qualitatively and in terms of T2 quantification. The 2D short axis views and the reformatted short axis views obtained with qBOOST‐T2 are shown in Figure 7 for 10 healthy subjects.

**Figure 8:**
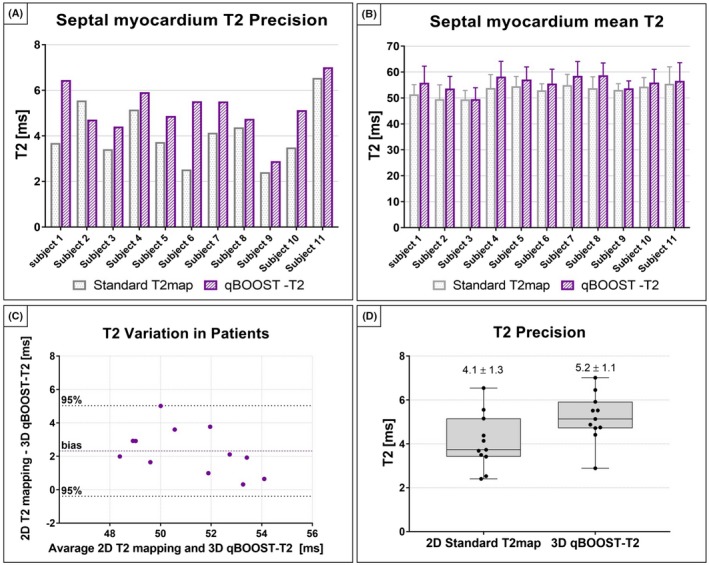
Quantification of septal myocardium mean T2 and precision of the proposed qBOOST‐T2 technique and comparison with conventional 2D T2 mapping. A, Comparison between myocardial mean T2 obtained with conventional 2D T2 mapping (gray) and the proposed 3D qBOOST‐T2 mapping sequence (blue) for each healthy subject. Good agreement is observed in terms of mean T2 between the 2 approaches. B, Comparison between myocardial T2 precision (measured as standard deviation with in a septal ROI) obtained with conventional 2D T2 mapping (gray) and the proposed 3D qBOOST‐T2 mapping sequence (blue) for each healthy subject. C, Bland Altman plot comparing the proposed qBOOST‐T2 sequence with the conventional 2D bSSFP T2 mapping technique. Good agreement is observed between the 2 approaches. A slight T2 overestimation is obtained with qBOOST‐T2mapping (bias = 2.71 ms), however, T2 quantification is within the 95% interval. D, Comparison between precision obtained with standard T2 mapping and the proposed qBOOST‐T2. A slightly lower (not significant) precision is observed with the proposed qBOOST‐T2 sequence. Myocardial T2 accuracy and precision were measured in a ROI in the septum of the myocardium

Quantitative analysis was carried out for all healthy subjects. Mean T2 value and standard deviation measurements in a septal ROI are shown in Figure 8. Good agreement with linear correlation y = 1.02x + 1.46 (R^2^ = 0.61) was found between the conventional 2D bSSFP T2 mapping and qBOOST‐T2. The average T2 values obtained with 2D bSSFP and 3D qBOOST‐T2 sequences were T2 = 53 ± 2 ms and T2 = 55 ± 3 ms, respectively. A statistically significant (*P* = 0.0003) slight overestimation in T2 quantification was observed with the proposed method. The proposed approach showed a slightly lower precision with respect to the standard 2D T2 mapping technique (4.09 ± 1.25 ms and 5.19 ± 10.9 ms for standard T2 mapping and qBOOST‐T2, respectively); however, it was not statistically significant. T2 quantification obtained with standard 2D bSSFP T2 mapping and qBOOST‐T2 mapping were also compared in a Bland‐Altman analysis (Figure 8C). A mean difference of 2.71 ms was observed between the 2 mapping techniques and the limits of 95% agreement were 0.61 ms and 6.03 ms.

**Figure 9:**
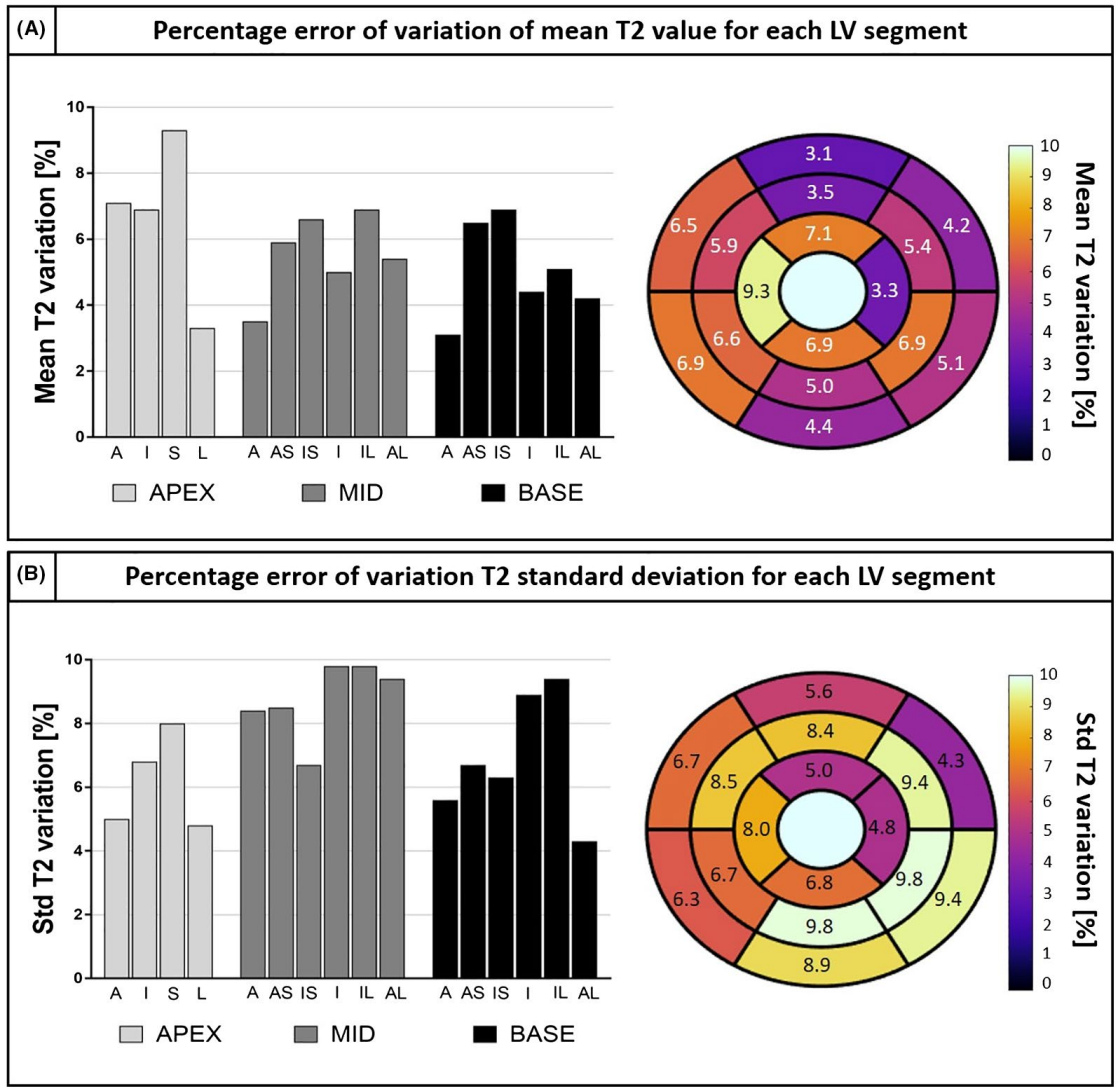
Percentage of variation of mean T2 (A) and T2 precision (B) between 2D bSSFP and 3D qBOOST‐T2. T2 overestimation and a lower precision are observed in each segment of the left ventricle. A, anterior; S, septal; I, inferior; L, lateral; AS, anterior‐septal; IS, inferior‐septal; IL, inferior‐lateral; AL, anterior‐lateral

Bar and bull's eye plots of the percentage of variation of mean T2 value and T2 standard deviation are shown in Figure 9. An overestimation of T2 is obtained with qBOOST‐T2 approach with respect to conventional 2D bSSFP in all left ventricular segments. Additionally, a lower precision is observed, especially in the inferior part of the left ventricle. However, precision may be affected not only by the different sequences but also by different imaging parameters, such as slice thickness and resolution. The effect of averaging contiguous 3D qBOOST‐T2 slices on precision has been investigated and the results are shown in Supporting Information Figure S2. Similar findings were obtained by investigating the effect of image resolution on T2 quantification (Supporting Information Figure S3).

### 3.4 Patients

**Figure 10:**
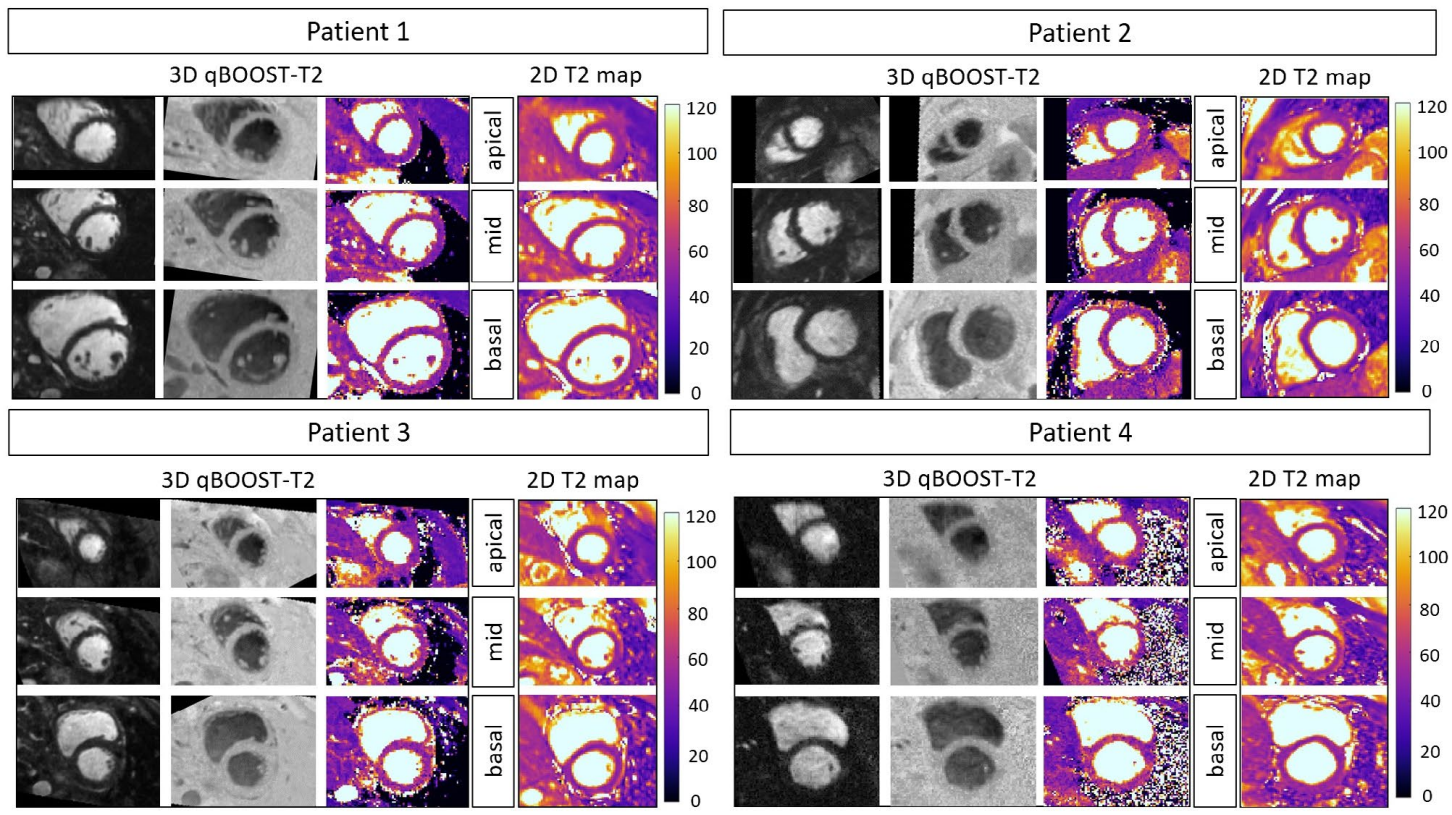
Comparison between 2D short‐axis standard T2 maps and short‐axis reformatted 3D qBOOST‐T2 maps for 4 patients with suspected cardiovascular disease. Apical, mid, and basal slices are shown for the acquired patients. Additionally, bright‐blood and black‐blood short axis reformatted images are shown for the qBOOST‐T2 acquisition. No pathologies were diagnosed for any of the acquired patients

The average scan time for the proposed qBOOST‐T2 was 10 min and 35 s. Bright‐ and black‐blood images, and T2 maps reformatted in coronal orientations are shown in Figure 10. Corresponding conventional 2D bSSFP T2 maps are also included in Figure 10 for comparison purposes. Myocardial septal T2 values were measured in apical, mid and basal slices for each subject and the results are reported in Supporting Information Table S3. A general overestimation (bias of 2.3 ms) and lower precision with respect to conventional 2D bSSFP T2 mapping was observed with the proposed approach. Bull's eye plot of mean myocardium T2 quantification and T2 standard deviation are shown in Supporting Information Figure S4A for a representative patient. A histogram of per‐pixel T2 distribution is shown in Supporting Information Figure S4B. The mean and standard deviation of T2 distribution were 46.5 ms and 6.8 ms, respectively. T2 distribution through coronal slices showed a linear correlation of y = −0.03x + 47.3 (Supporting Information Figure S4C).

## 4 DISCUSSION

In this study, a 3D free‐breathing accelerated qBOOST‐T2 sequence for simultaneous and co‐registered acquisition of anatomical high‐resolution bright‐blood and black‐blood volumes and a 3D T2 map has been proposed.

This approach was based on the acquisition of three 4× undersampled interleaved bright‐blood whole‐heart datasets acquired with different magnetization preparations: (1) T2prep‐IR preparation module, (2) T2 preparation, and (3) no preparation. The T2prep‐IR prepared dataset provided bright‐blood anatomical visualization, the black‐blood volume was obtained by performing a PSIR reconstruction between the first and third dataset and the 3D T2 map was generated by matching the acquired signal evolution to a dictionary obtained by means of EPG simulations. The use of 2D image‐based navigators allowed SI and LR translational motion correction with 100% respiratory scan efficiency and predictable scan time. Whereas, the use of a 3D patch‐based PROST reconstruction enabled 4× undersampled acquisition preserving good visual image quality.

The proposed qBOOST‐T2 has been designed to enable a comprehensive assessment of the heart including anatomical visualization and T2 myocardial tissue quantification in a single free‐breathing scan, thus overcoming some of the limitations of current sequential acquisitions, such as misalignment and long scan times. The 3D acquisition allowed whole‐heart myocardium coverage in comparison to conventional breath‐hold 2D T2 mapping maintaining uniform T2 quantification across the whole left ventricle and across different slices. Additionally, the nearly isotropic high‐resolution nature of the acquisition permitted to reformat the co‐registered bright‐blood, black‐blood volumes, and T2 maps in different orientations (coronal, transversal, short‐axis, and 4‐chamber) preserving good image quality and uniform T2 quantification in a clinically feasible scan time, in comparison to recently proposed 3D T2 mapping methods with lower resolution that do not allow reformatting the 3D volume in different orientations^35^, ^36^ or that requires long acquisition time.^37^

The proposed qBOOST‐T2 approach showed good accuracy and precision with respect to spin echo reference values (high linear correlation) in the phantom experiment. T2 quantification was found to be robust to T1 variability in both simulations and phantom experiments (T2 variability <5%). Sequence simulations showed robustness to different HRs (percentage of variation <5%). Higher dependency on HR was observed in the phantom scan (variability of 10%); however, capability to differentiate between different T2 values ranging between 25 ms and 115 ms was observed.

Good delineation of anatomical structures was observed in the bright‐blood volume acquired with qBOOST‐T2 approach. However, lower sharpness was observed in the reformatted coronary qBOOST‐T2 image (Figure 6), which may be caused by the undersampled nature of the acquisition and by residual motion that could affect fine resolution details.

Good agreement in terms of T2 quantification was observed between T2 maps obtained with the proposed qBOOST‐T2 sequence and standard bSSFP T2 mapping in healthy subjects. A slight T2 overestimation and a lower precision was observed with the proposed approach in comparison to conventional 2D bSSFP T2 mapping. However, the difference in measured precision was not statistically significant. The bias in T2 quantification between qBOOST‐T2 and 2D bSSFP T2 mapping calculated with the Bland‐Altman analysis was 2.7 ms (within the limits of 95%). The slight T2 overestimation of qBOOST‐T2 with respect to 2D bSSFP T2 mapping was likely due to the different k‐space ordering used by both sequences (centric for qBOOST‐T2 and linear for conventional 2D T2 mapping), as has been reported before.^3^ Whereas, the high‐resolution 3D nature of qBOOST‐T2 (slice thickness = 2 mm) may explain the lower precision observed with the proposed approach with respect to 2D T2 mapping (slice thickness = 8 mm). A trade‐off between image resolution, T2 precision, and partial volume have been observed (Supporting Information Figures S2 and S3); thus, the lower precision observed in the in vivo experiment may not only be due to the proposed technique but due to different image parameters adopted in the 3D and 2D scans (i.e., resolution). Our experiments showed that decreasing the resolution leads to an increased precision, associated to the increased signal to noise ratio in each acquired volume and thus an increased precision. However, it has been previously shown^20^ that low‐ resolution acquisitions may introduce partial volume artefacts that could affect T2 quantification and precision.

A general T2 overestimation across the whole myocardium with respect to conventional 2D bSSFP T2 mapping was also observed in the bull's eye plots. However, T2 quantification was uniform across the whole 3D volume. A lower precision was observed particularly in the inferior part of the heart, which may be explained by the presence of residual motion in the reconstructed images and lower signal to noise ratio due to larger distance to the radiofrequency coils. Additionally, the inferior region of the heart is located close to the edge of the FOV; thus, imperfect shimming could lead to field inhomogeneities that would affect the T2 map. Moreover, a lower signal to noise is expected in the qBOOST‐T2 acquisition due to lower slice thickness.

Preliminary results in 4 patients showed a similar trend as noticed in the healthy subject study. A slight T2 overestimation was observed in each acquired short‐axis slice when compared with standard 2D bSSFP T2 mapping, with slightly lower precision. However, the 3D whole heart coverage of the proposed approach provides the flexibility to reformat the acquired volume in any orientation, which could be beneficial for the identification of localized pathologies as shown in van Heeswijk et al.^37^

A potential limitation of the proposed work is fat suppression. Different fat suppression techniques are used on each acquired dataset because fat signal evolution differs in each volume. In the first dataset, a STIR approach is used to achieve fat suppression. The inversion pulse of the T2prep‐IR module was used to null the fat signal with an TI of 110 ms. In the second and third dataset, a SPIR approach was used and spectral presaturation FAs of 110 degrees and 130 degrees were used to null fat signal in the second and third volume, respectively. Both TI and SPIR FAs were optimized for a HR of 60 bpm, however, the HR dependency of fat suppression techniques could lead to residual fat signal in 1 or more reconstructed volumes. If a suboptimal fat suppression is achieved in 1 or more of the acquired volumes an unpredictable signal will be matched in the T2 map: depending on the acquired signal evolution, the T2 corresponding to the closest dictionary entry to the measured signal will be matched. Moreover, residual fat signal could generate partial volume artefacts thus affecting the T2 quantification at the myocardium‐fat interface. In the presence of partial volume, the mixed signal will be matched to the closest signal evolution entry in the dictionary; however, it will not reflect the proper T2 value of the voxel. The least square error of the matching process could be used to assess the accuracy of the matching in the presence of partial volume artefacts.

The approximation of the standard deviation of the proposed technique used in this study ignores intrinsic variability of underlying T2 because uniform mean T2 values were expected across healthy subjects. However, this approximation is valid only when analyzing normal T2 values and percentages of the mean should be considered in future patient studies.

An additional limitation of the proposed technique is the approximation of respiratory motion as pure translational motion in SI and LR directions. Respiration induces additional displacements of the heart such as translational motion in the anterior‐posterior direction, as well as rotation and nonrigid deformation.^38^, ^39^, ^40^ Future studies will focus on the implementation and optimization of nonrigid respiratory motion correction within the reconstruction.^41^

A further limitation is the sensitivity to arrhythmia. In the presence of arrhythmia, the measured signal would differ from the steady state signal expected in the 3 different interleaved acquisitions, generating a T2 overestimation or underestimation in the matched T2 maps. Prospective or retrospective arrhythmia rejection could be incorporated in the future to overcome this limitation. With a prospective arrhythmia rejection approach, 3 interleaved beats will be rejected in the presence of 1 arrhythmic heart beat and the entire acquisition will be repeated with a stabilized HR; however, this approach will lead to longer and unpredictable acquisition time. On the other hand, by exploiting retrospective arrhythmia rejection, all the datasets will be acquired and the data corrupted by arrhythmic heart beats will be excluded from the reconstruction. However, the reconstructed dataset will be further undersampled (an undersampling factor of 4 is used to accelerate the acquisition); thus, in the presence of high undersampling, the image quality of the reconstructed datasets and, therefore, of the matched T2 maps may be compromised. Validation of the proposed approach in patients with cardiovascular disease and challenging acquisition conditions, i.e., arrhythmic heart beat will be investigated in future studies.

## 5 CONCLUSIONS

The proposed accelerated qBOOST‐T2 sequence allows the acquisition of 3D co‐registered high‐resolution bright‐ and black‐blood volumes and T2 map for comprehensive assessment of cardiovascular disease in a clinically feasible scan time of ~11 min. The proposed approach shows promising results in terms of accurate T2 quantification when compared with conventional 2D T2 mapping. Future work will include further validation in patients with cardiovascular disease.

## Supporting information


**FIGURE S1** EPG simulations performed to assess the matched T2 dependency on the T1 used to generate the simulated signal. A, Signal evolution of T1/T2 pairs with T2 = (40:6:88) ms and T1 = (800:100:1400) ms were matched to a EPG dictionary with fixed T1 = 1100 ms. High T1 dependency was observed for long T2 values. B, Signal evolution of T1/T2 pairs with T2 = (40:6:88) ms and T1 = (800:100:1400) ms were matched to a EPG dictionary with T1 = (900, 1100, 1300) ms. T2 matching percentage error was decreased and a T2 variation < 5% was observed for almost all the simulated signal. C, Maximum variability errors (T1 = 800 and 1400 ms) obtained by matching the simulated signal to a dictionary with fixed T1 (top row) and a dictionary with T1 = (900, 1100, 1300) ms (bottom row)
**FIGURE S2** A, Effect of averaging contiguous slice on T2 quantification and T2 precision. Averaging 6 contiguous slices leads to a reduction of standard deviation in a septal ROI from 5.90 ms to 3.39 ms (percentage of variation of 42.5%), whereas no effect on T2 quantification was observed (T2 variability of only 1.1%). B, T2 intensity profile drawn across a septal region (indicated by the black line) for different number of summed slices. Decreasing resolution in the slice direction leads to an increase of partial volume effects between blood and myocardium. Indeed, a narrower myocardial delineation is observed for a high number of summed slices. Additionally, partial volumes effects are visible in lower resolution images as shown by the black arrow
**FIGURE S3** A, Three 3D qBOOST‐T2 maps were generated for 1 representative subject with reconstructed resolutions of 1 × 1 × 2 mm^3^, 1.5 × 1.5 × 3 mm^3^ and 2 × 2 × 4 mm^3^ and compared with 2D bSSFP T2 map. B, Mean T2 and T2 precision measured in the septum of the myocardium as function of different reconstructed resolutions for 3D qBOOST‐T2. A reduction in standard deviation is observed, whereas a variability of only 0.96% in myocardial T2 quantification was observed between different resolutions. Table: Mean and standard deviation of T2 measured in the septum for different reconstructed resolutions
**FIGURE S4** A, Bull's eye plot of averaged myocardial T2 quantification and precision of the proposed qBOOST‐T2 mapping sequence for patient 2. B, Histogram of per‐pixel T2 distribution through the whole left ventricle. C, Averaged T2 distribution through coronal slices showed a linear correlation of y = −0.03x + 47.3. Uniform T2 quantification is observed in the left ventricle
**TABLE S1** T1 and T2 values obtained from Inversion Recovery Spin Echo (IRSE) and Spin Echo (SE) experiments on a phantom with 6 vials with different agar concentration (0.8, 1, 1.5, 2, 3, 5%). The measured T2 values are within a range than includes T2 of physiological and pathological myocardium (T2_myoc_ = 52 ms T2_myoc‐diaseased_ = 65 ms)
**TABLE S2** Acquisition parameters used in phantom and in vivo acquisition for 2D bSSFP T2 mapping, 3D qBOOST‐T2 and coronary magnetic resonance angiography (CMRA)
**TABLE S3** Measured septal myocardial T2 values obtained with qBOOST‐T2 and conventional 2D bSSFP for 4 patients. A general T2 overestimation and lower precision is observed with the proposed technique

**VIDEO S1** Bright‐blood 3D volume acquired with qBOOST‐T2 for a representative healthy subject

**VIDEO S2** Co‐registered black‐blood 3D volume acquired with qBOOST‐T2 for same healthy subject shown in Video S1

**VIDEO S3** Co‐registered 3D T2 map acquired with qBOOST‐T2 for same healthy subject shown in Videos S1 and S2. Uniform T2 quantification is observed across the whole myocardium
