# Supplementary material for: 3D Whole‐heart free‐breathing qBOOST‐T2 mapping
Source: Magn Reson Med. 2019 Oct 21;83(5):1673–87. doi: 10.1002/mrm.28039 (PMC7004111; doi:10.1002/mrm.28039)
Supplement: Supplementary file 1 — FIGURE S1 EPG simulations performed to assess the matched T2 dependency on the T1 used to generate the simulated signal. A, Signal evolution of T1/T2 pairs with T2 = (40:6:88) ms and T1 = (800:100:1400) ms were matched to a EPG dictionary with fixed T1 = 1100 ms. High T1 dependency was observed for long T2 values. B, Signal evolution of T1/T2 pairs with T2 = (40:6:88) ms and T1 = (800:100:1400) ms were matched to a EPG dictionary with T1 = (900, 1100, 1300) ms. T2 matching percentage error was decreased and a T2 variation < 5% was observed for almost all the simulated signal. C, Maximum variability errors (T1 = 800 and 1400 ms) obtained by matching the simulated signal to a dictionary with fixed T1 (top row) and a dictionary with T1 = (900, 1100, 1300) ms (bottom row) FIGURE S2 A, Effect of averaging contiguous slice on T2 quantification and T2 precision. Averaging 6 contiguous slices leads to a reduction of standard deviation in a septal ROI from 5.90 ms to 3.39 ms (percentage of variation of 42.5%), whereas no effect on T2 quantification was observed (T2 variability of only 1.1%). B, T2 intensity profile drawn across a septal region (indicated by the black line) for different number of summed slices. Decreasing resolution in the slice direction leads to an increase of partial volume effects between blood and myocardium. Indeed, a narrower myocardial delineation is observed for a high number of summed slices. Additionally, partial volumes effects are visible in lower resolution images as shown by the black arrow FIGURE S3 A, Three 3D qBOOST‐T2 maps were generated for 1 representative subject with reconstructed resolutions of 1 × 1 × 2 mm3, 1.5 × 1.5 × 3 mm3 and 2 × 2 × 4 mm3 and compared with 2D bSSFP T2 map. B, Mean T2 and T2 precision measured in the septum of the myocardium as function of different reconstructed resolutions for 3D qBOOST‐T2. A reduction in standard deviation is observed, whereas a variability of only 0.96% in myocardial T2 quantification was obs [file MRM-83-1673-s001.docx]

**Supporting Information** **Figures**


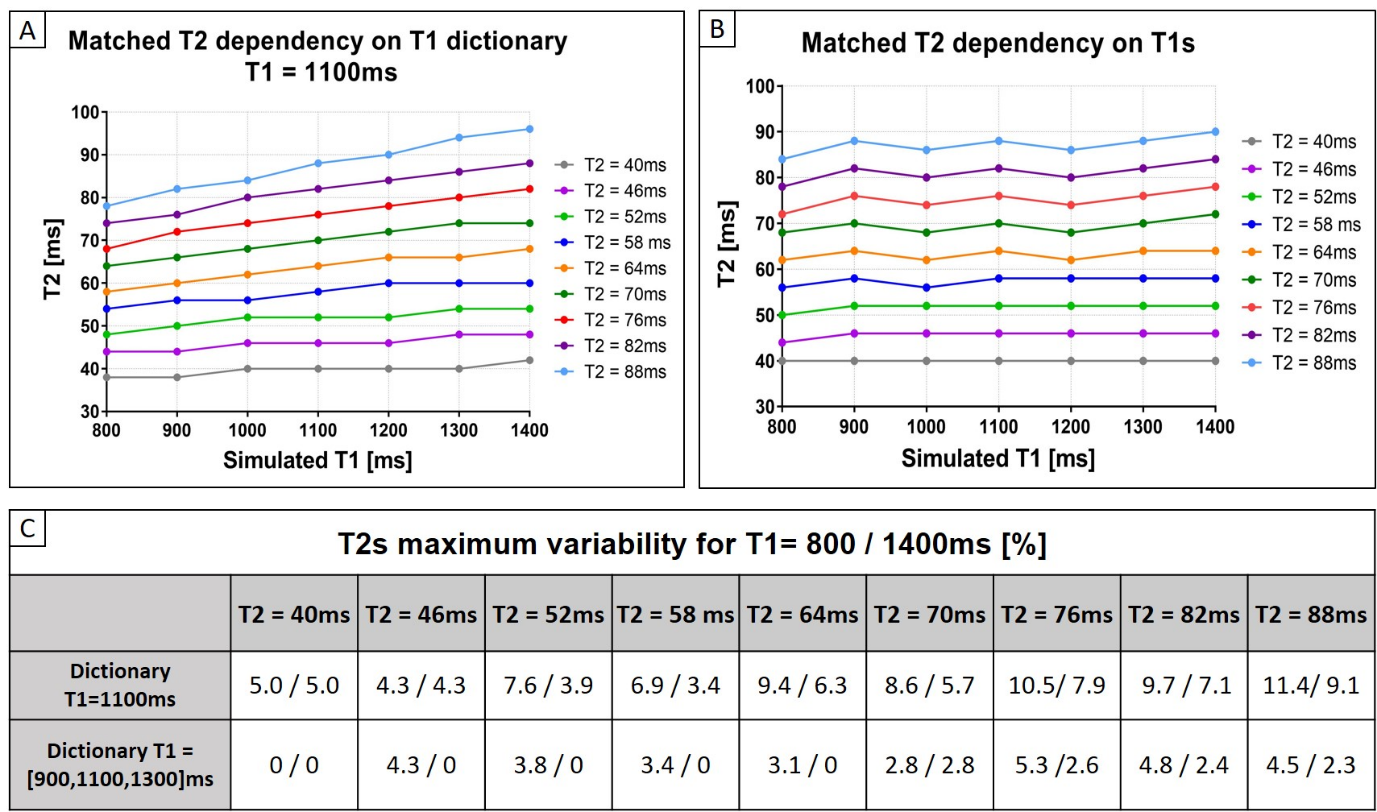


Supporting Information Figure S1 - EPG simulations performed in order to assess the matched T2 dependency on the T1 used to generate the simulated signal. A) Signal evolution of T1/T2 pairs with T2=[40:6:88]ms and T1=[800:100:1400]ms were matched to a EPG dictionary with fixed T1=1100ms. High T1 dependency was observed for long T2 values. B) Signal evolution of T1/T2 pairs with T2=[40:6:88]ms and T1=[800:100:1400]ms were matched to a EPG dictionary with T1=[900, 1100, 1300]ms. T2 matching percentage error was decreased and a T2 variation < 5% was observed for almost all the simulated signal. C) Maximum variability errors (T1=800 and 1400ms) obtained by matching the simulated signal to a dictionary with fixed T1 (top row) and a dictionary with T1=[900, 1100, 1300]ms (bottom row).


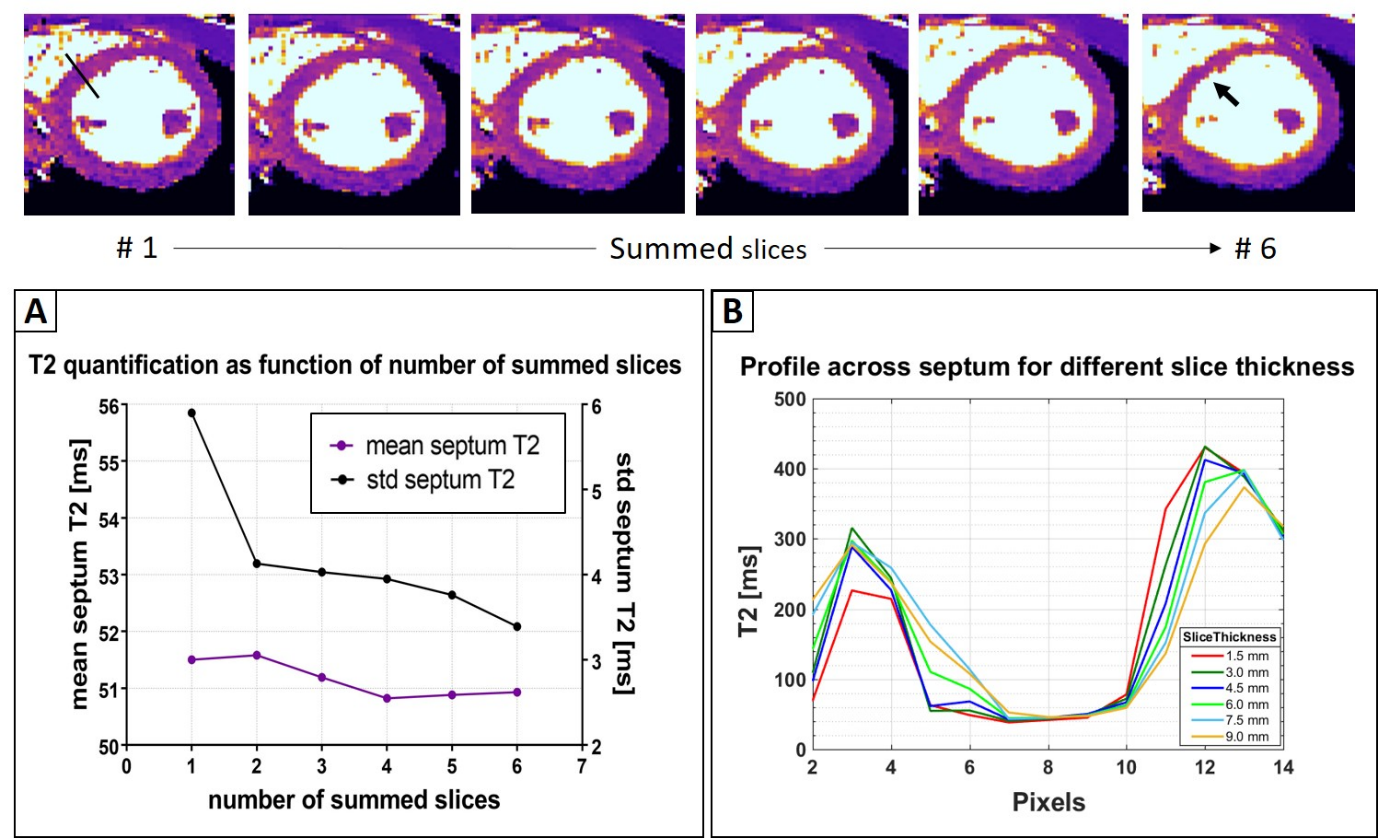


Supporting Information Figure S2 - A) Effect of averaging contiguous slice on T2 quantification and T2 precision. Averaging six contiguous slices leads to a reduction of standard deviation in a septal ROI from 5.90ms to 3.39ms (percentage of variation of 42.5%), whereas no effect on T2 quantification was observed (T2 variability of only 1.1%). B) T2 intensity profile drawn across a septal region (indicated by the black line) for different number of summed slices. Decreasing resolution in the slice direction leads to an increase of partial volume effects between blood and myocardium. Indeed, a narrower myocardial delineation is observed for a high number of summed slices. Additionally partial volumes effects are visible in lower resolution images as shown by the black arrow.


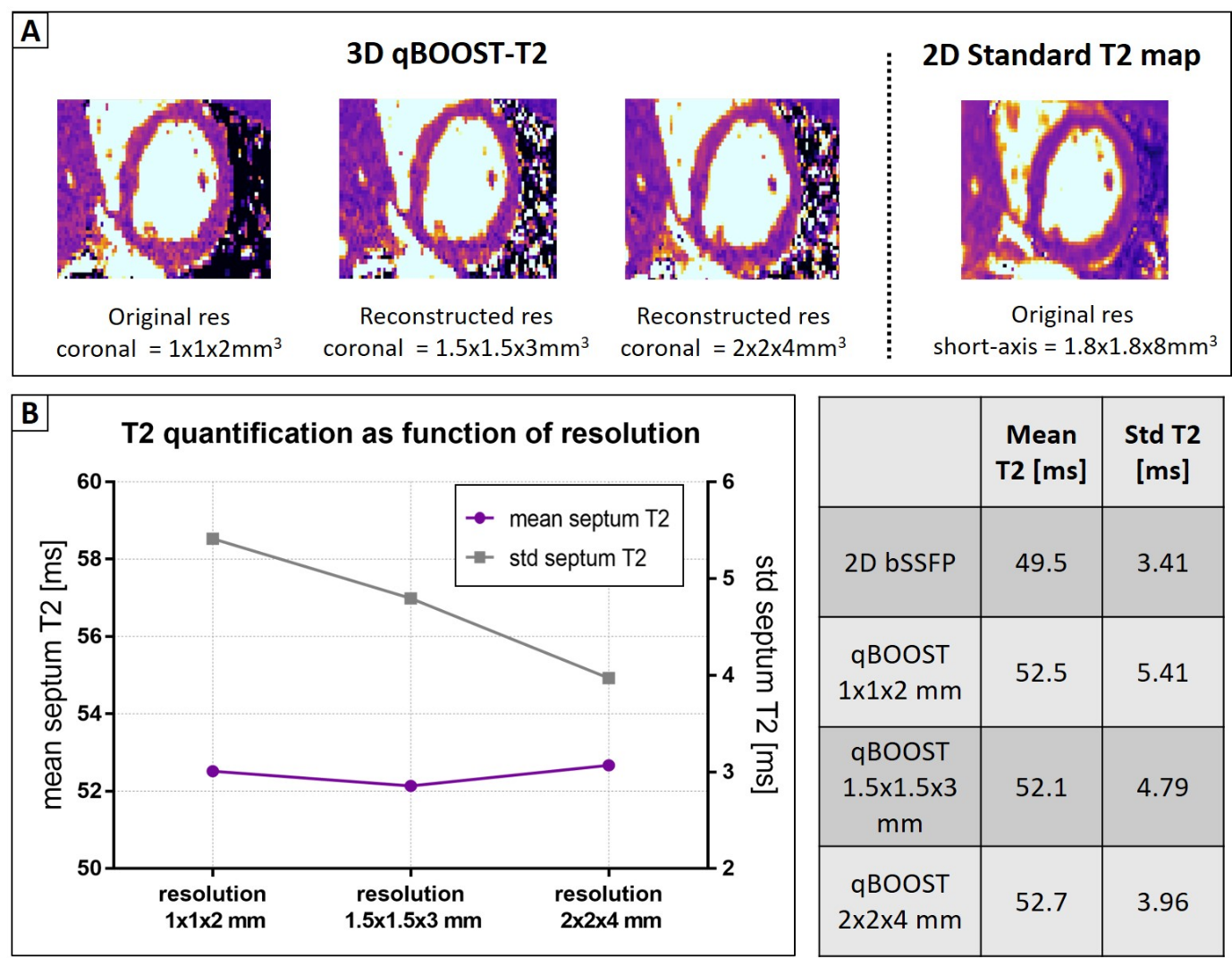


Supporting Information Figure S3 - A) Three 3D qBOOST-T2 maps were generated for one representative subject with reconstructed resolutions of 1x1x2mm3, 1.5x1.5x3mm3 and 2x2x4mm3 and compared to 2D bSSFP T2 map. B) Mean T2 and T2 precision measured in the septum of the myocardium as function of different reconstructed resolutions for 3D qBOOST-T2. A reduction in standard deviation is observed, whereas a variability of only 0.96% in myocardial T2 quantification was observed between different resolutions. Table: Mean and standard deviation of T2 measured in the septum for different reconstructed resolutions.


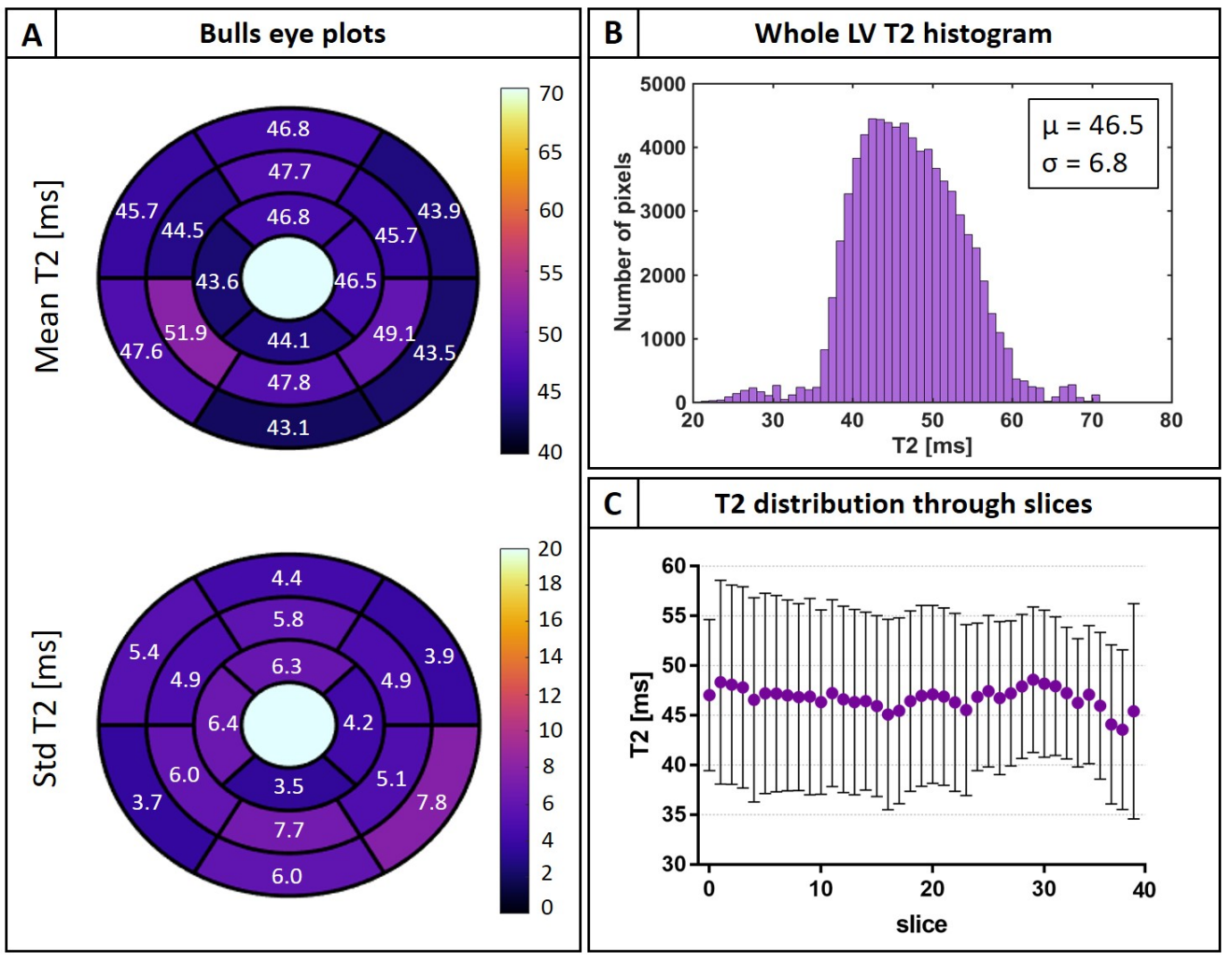


Supporting Information Figure S4 - A) Bull’s eye plot of averaged myocardial T2 quantification and precision of the proposed qBOOST-T2 mapping sequence for patient 2. B) Histogram of per-pixel T2 distribution through the whole left ventricle. C) Averaged T2 distribution through coronal slices showed a linear correlation of y = -0.03x + 47.3. Uniform T2 quantification is observed in the left ventricle.


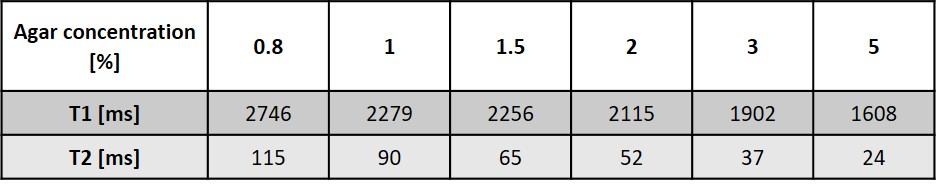


Supporting Information Table S1 - T1 and T2 values obtained from Inversion Recovery Spin Echo (IRSE) and Spin Echo (SE) experiments on a phantom with six vials with different agar concentration (0.8, 1, 1.5, 2, 3, 5%). The measured T2 values are within a range than includes T2 of physiological and pathological myocardium (T2myoc = 52ms T2myoc-diaseased = 65ms).


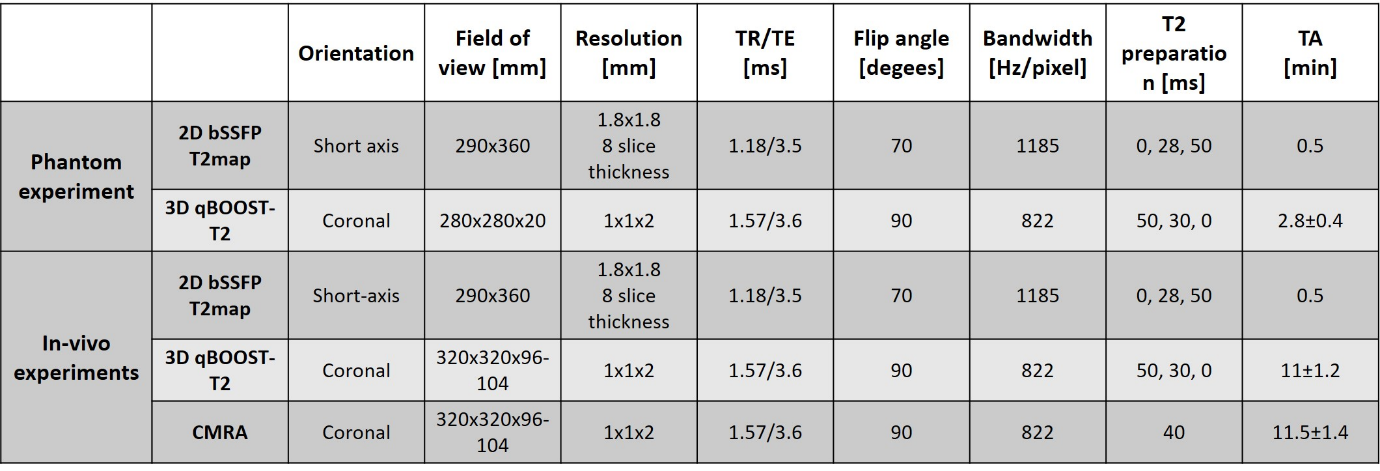


Supporting Information Table S2 - Acquisition parameters used in phantom and in-vivo acquisition for 2D bSSFP T2 mapping, 3D qBOOST-T2 and coronary magnetic resonance angiography (CMRA).


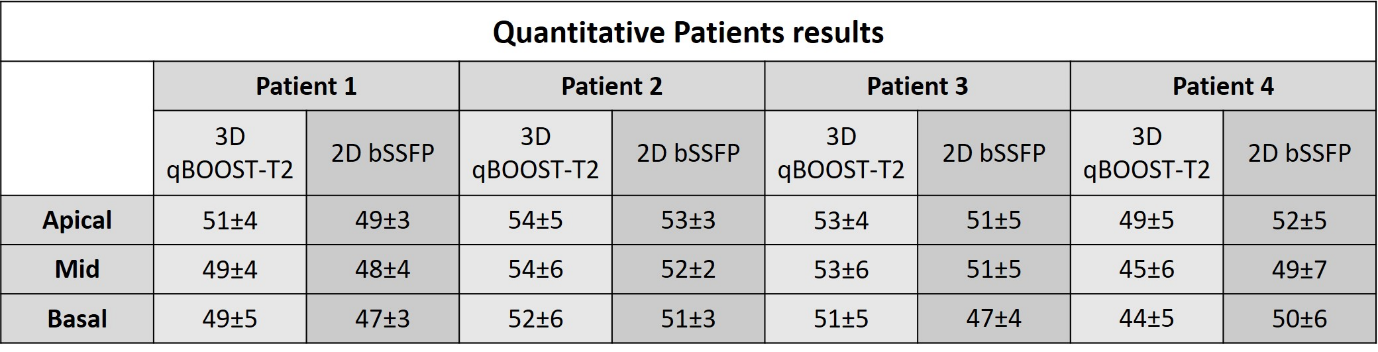


Supporting Information Table S3 - Measured septal myocardial T2 values obtained with qBOOST-T2 and conventional 2D bSSFP for four patients. A general T2 overestimation and lower precision is observed with the proposed technique.
