# Supplementary figures and images for: 3D Whole‐heart free‐breathing qBOOST‐T2 mapping
Source: Magn Reson Med. 2019 Oct 21;83(5):1673–87. doi: 10.1002/mrm.28039 (PMC7004111; doi:10.1002/mrm.28039)

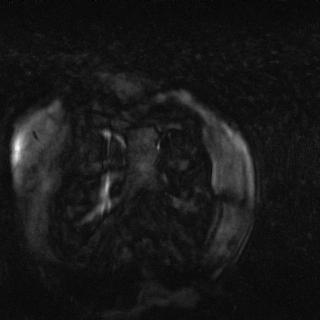

Supplement: Supplementary file 2 — VIDEO S1 Bright‐blood 3D volume acquired with qBOOST‐T2 for a representative healthy subject [file MRM-83-1673-s002.gif]

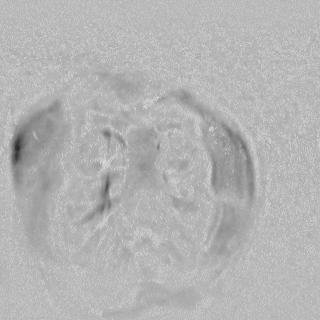

Supplement: Supplementary file 3 — VIDEO S2 Co‐registered black‐blood 3D volume acquired with qBOOST‐T2 for same healthy subject shown in Video S1 [file MRM-83-1673-s003.gif]

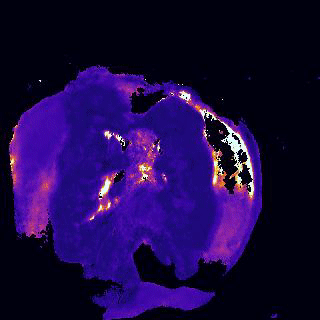

Supplement: Supplementary file 4 — VIDEO S3 Co‐registered 3D T2 map acquired with qBOOST‐T2 for same healthy subject shown in Videos S1 and S2. Uniform T2 quantification is observed across the whole myocardium [file MRM-83-1673-s004.gif]
